# Knowledge, attitude, practice and associated factors towards spectacles use among adults in Gondar town, northwest Ethiopia

**DOI:** 10.1186/s12886-016-0357-3

**Published:** 2016-10-21

**Authors:** Alemayehu Desalegn, Asamer Tsegaw, Destaye Shiferaw, Haile Woretaw

**Affiliations:** 1 Department of Optometry, College of Medicine and Health Science, University of Gondar, Gondar, Ethiopia; 2 Department of Ophthalmology, College of Medicine and Health Science, University of Gondar, Gondar, Ethiopia

**Keywords:** Spectacles, Knowledge, Attitude, Practice, Gondar

## Abstract

**Background:**

Refractive error is the main cause of visual impairment in the world. Spectacles are the most frequently used options for correcting refractive errors. In addition, they can be used for protection and fashion. It is the simplest, cheapest and only method used in developing countries like Ethiopia. This study aims to explore the knowledge, attitude, practice and associated factors towards spectacles use among adult population of Gondar town, northwest Ethiopia.

**Methods:**

Community based cross sectional study was conducted on 780 participants using a pre-tested structured questionnaire in Gondar. Data were analyzed using Statistical Package for Social Sciences (SPSS) 20 version computer software. Association and strength between variables was determined using odds ratio with 95 % confidence interval.

**Result:**

A total of 780 study subjects participated in this study. The male to female ratio was 1:2.4. The median age of the participants was 29 (±22 IQR) with a range of 18–86 years. About fifty percent of participants were married and 284 (36.4 %) were educated up to secondary school. Seven hundred and three (90.6 %) participants had adequate knowledge about spectacles and 90.4 % had favorable attitude towards spectacle use. About 25 % of the participants have been using spectacles during the study. Participants with primary school education (AOR: 2.79, 95 % CI 1.20–6.50) had good knowledge about spectacles. Housewives (AOR = 3.40, 95 % CI; 1.35–8.54) and participants who unable to read and write (AOR: 3.51, 95 % CI 14–10.72) had favorable attitude towards spectacles use.

**Conclusion:**

Gondar town adult population has adequate knowledge and favorable attitude towards spectacles. However, practice of spectacles use is poor. Eye health education related to spectacles utilization need to be given due emphasis by eye care professionals in collaboration with University of Gondar and Gondar town administration.

## Background

Spectacles are optical instruments consisting of a frame that holds a pair of lenses to correct defective visions (reading and/or distance), protection, fashion and for achievement of confidence [1–3].

Refractive error is a common cause of visual impairment and blindness and It is estimated that 2.3 billion people lives with refractive errors worldwide [4, 5]. There are an estimated 153 million people with visual impairment due to uncorrected refractive errors, i.e. presenting visual acuity < 6/18 in the better eye, excluding presbyopia [6]. The prevalence of refractive errors varies from one place to the other ranging from 20 to 80.5 % [7, 8].

Uncorrected refractive errors are increasingly being addressed in national plans for the prevention of blindness, and low-cost, good-quality spectacles are becoming available [6].

Despite the fact that, majority of those with refractive errors could have their sight restored with spectacles, it is only 1.8 billion have access to eye examinations and affordable correction. This leaves approximately 500 million people, mostly in developing countries (close to one-third are in Africa) and many children, with uncorrected refractive error which exposes them to blindness and impaired vision. Many are not aware that there is a correction for their compromised vision, have no one to provide treatment, or cannot afford the appliances they need. In the above 50 years, at least 80 % of them require spectacle corrections for near vision [9].

The way to eliminate uncorrected refractive error is through the development of all aspects of a self-sustaining system, including human resources to provide eye care services; and spectacles to correct vision [5]. Spectacles are the most frequently used options for correcting refractive errors as they are the simplest, cheapest and most widely used methods that have a high success rate in terms of visual acuity, quality of life and cultural acceptance to rural as well as urban populations [6].

Though spectacles has been included in the essential drug list of the World Health Organization’s [10], people are not using glasses even when prescribed by a specialist due to beliefs and attitudes of users, parent (if children) and the community as a whole [2, 3].

It was vision 2020’s plan to improve public awareness, generate demand for services through community-based initiatives, primary eye care, and school eye-health programmers. Specifically, in low-income settings, provided that spectacles are new, of good quality, accessible and affordable [6].

This study explored knowledge, attitude, practice and associated factors towards spectacles use among adult population in Gondar. The findings will help efforts to be geared towards altering them or strengthening them. It will also give base line information for further study to be conducted on similar topics.

## Methods

Community based cross sectional study was conducted in Gondar town, northwest Ethiopia, Amhara National Regional State, 738 Km from the capital city (Addis Ababa) and 180 Km from Bahir Dar (Capital city of Amhara National Regional State). According to the 2007 national central statistical agency, an estimated population of 207,044 lives in the town of whom 98,120 and 108,924 male and female respectively with total house hold count of 53,725. Gondar town has 24 administrative areas (kebeles). There is one tertiary eye care and training center in the town, which serves more than 14 million people of the catchment area. In the eye, care and training center there is one optical workshop where spectacles are glazed and fitted. The study was conducted from November 2013 to December 2013.

Adults age ≥18 years old living in Gondar town were the source population. The inclusion criterion was permanent residence in Gondar town. Individuals who were unable to respond to the questionnaire due to either serious illness or mental problem and those who resided less than 6 month in the study area were excluded.

Sample size was determined by using the single population proportion formula. Considering the proportion of people with adequate knowledge, favorable attitude and good practice towards spectacles use 50 %, a design effect of 2 and 10 % of non-response rate, the final sample size was 845. (Detail annexed)

In order to reach the calculated sample, a multistage random sampling technique was used. Initially, 4 kebeles (local administrative areas) out of 12 were selected using computer generated random numbers after a census list of all kebeles. Households were allocated for each kebele proportionally. Systematic random sampling was employed to select households (with sampling fraction of constant K = 14). Finally, lottery method was used to select participants in households where more than one adult (≥18 years old) was present.

The outcome variables were knowledge (adequate/inadequate), attitude (favorable/unfavorable) and practice (good/poor).

Operational definition: favorable attitude: those respondents who responded mean and above to attitude questionnaires toward spectacles; unfavorable attitude: those respondents who responded below the mean to the attitude questionnaires towards spectacles; knowledge about spectacles: individual who responded mean and above of the total knowledge questionnaire had adequate knowledge about spectacles and those scoring below the mean had inadequate knowledge about spectacles; practice of spectacle use: good practice for those respondents who wear spectacles greater than or equal to fifty percent of the time for intended use and poor practice for those respondents who wear spectacles below fifty percent of the time for intended use.

Face to face interview was employed using structured questionnaires to collect data from 2^nd^ November to 24^th^ November 2013. Eight hundred forty-five households were selected through systematic random sampling technique. First, data collectors (six optometrists and eight 4th year optometry students) introduced themselves and explained the purpose of the study. Oral informed consent was obtained from each participant. If there was no individual in the selected household on the first visit of data collection, the house was re-visited. Non-response was considered when the randomly selected individual was not willing to participate and the reason for refusal was recorded.

In order to ensure the quality of data, the questionnaire was translated from English to Amharic (local language) and then back to English by language expertise for consistency. Pre-test was conducted (on 5 % of the sample size) in Azezo, which was not included in the sample area, and training was given for data collectors for 1 day. Data were checked for completeness, clarity and cleanliness before analysis by the supervisor.

After coding, data were entered into EPI INFO 2002 and exported to and analyzed using SPSS version 20. Descriptive statistics was used to compute the proportion of knowledge, attitude and practice towards spectacles use. Binary logistic regression was carried out to identify factors associated with the outcome variables. Variables that were found to be association at *p*-value of 0.2 and below were entered into the multiple logistic regression. Odds ratio with 95 % confidence interval at *p* < 0.05 was used to determine statistically significant association.

## Results

### Socio-demographic characteristics of respondents

A total of 780 study subjects participated in the study. The response rate was 92.2 %. The male to female ratio was 1:2.4. Five hundred and fifty one (70.6 %) were females and 229 (29.4 %) were males. The median age of the participants was 29 (±22 IQR) with range of 18–86 years. Four hundred seventy eight (61.3 %) were between 18 and 34 years. Three hundred and ninety four (50.5 %) participants were married, 637 (81.7 %) were Christian Orthodox in religion, 736(94.4 %) were Amhara in ethnicity, 284 (36.4 %) were educated up to secondary school and 238(30.5 %) were house wives (Appendix, Table 1).

### Knowledge about spectacles among study participants

Among the total participants, 703 (90.6 %) of them had adequate knowledge about spectacles. Among participants with adequate knowledge, 209 (29.8 %) source of information was from colleagues. Six hundred and fifty nine (94.0 %) of participants responded that spectacles are useful of whom 397 (56.5 %) responded as spectacles are useful for both distances (near and distance), protection and fashion (Appendix, Table 2).

### Attitude towards spectacles use in the study population

From the total 780 respondents, 705 (90.4 %) agreed to use spectacles if prescribed by eye care professional whereas 75 (9.6 %) disagree. Worsening existing problem 18 (24.0 %), age 17 (22.4 %), social unacceptability 13 (17.3 %) were frequently mentioned as reasons for not wearing. Six hundred and eighty four (87.7 %) of the respondents agreed that spectacles can be worn at any age and 725 (92.9 %) of the respondents agreed that spectacles can be worn by male and female if prescribed by eye care professionals. Five hundred and fifty two (70.8 %) of participants also agreed that wearing spectacles which are not prescribed by eye care professionals can cause problem to the eye. Overall, 715(91.7 %) of the respondents had favorable attitude towards spectacles use (Appendix, Table 3).

### Practice of spectacles use in the study population

From the total 780 participants, 194 (24.9 %) have been using spectacles. The reasons mentioned for using spectacles include: near vision 51 (26.3 %), distance vision 13 (6.7 %), protection 65 (33.5 %) and fashion 35 (18.1 %). More than half (56.2 %) of the spectacles users got their spectacles without eye examination. Forty four (22.7 %), 68 (35.0 %), and 30 (15.5 %) of the participants got their spectacles from government service centers, shops and on streets respectively. About 34 % of spectacles users have good practice. Among previous spectacle users about 26 % do not use spectacles currently due to spectacles break and 29 % due to ignorance (Appendix, Table 4).

### Variables associated with knowledge about spectacles

Educational status has an overall statistical association with knowledge about spectacles (overall *P* < 0.001). Primary school level of education has statistically significant association with knowledge about spectacles (AOR = 2.79, 95 % CI, 1.20–6.50) (Appendix, Table 5).

### Variables associated with attitude towards spectacles use

Occupation (*p* < 0.05) and educational level (*p* < 0.0001) showed an overall statistical significant with attitude towards spectacle use. Among the occupations, housewife (AOR = 3.40, 95 % CI; 1.35–8.54) and among the educational levels unable to read and write (AOR = 3.51, 95 % CI; 14–10.72) were significantly associated with attitude towards spectacles use. Inadequate knowledge about spectacles is significantly associated (AOR = 8.25 95 % CI; 4.33–15.73) with attitude towards spectacles use (Appendix, Table 6).

### Variables association with spectacles use (practice)

Only age of the respondents showed association with practice of spectacles use. Age category 35–44 years (AOR = 0.20, 95 % CI; 0.04–0.93), 55–64 years (AOR = 0.18, 95 % CI; 0.04–0.84), and 65–74 years (AOR = 0.02, 95 % CI; 0.00–0.2) has an association with practice of spectacle use (Appendix, Table 7).

## Discussion

In this study, about 91 % of the participants had adequate knowledge about spectacles. This result is higher than study done in India [11]. This might be due to the difference in the study setting; the study in India was rural setting while this study was conducted in urban setting. People living in urban have multiple source of information to know about spectacles: health centers, mass media and higher people interactions than those live in rural. Again, the proportion of educated people living in urban may be higher than those live in rural, which attributed to knowledge about spectacles.

The most frequently reported source of information for knowledge about spectacles was colleagues. This might be because people-to-people interaction (those wearing spectacles and not wearing) was ease way to get knowledge about spectacles than other source of information in the study area.

More than half (56.5 %) of the respondents reported that spectacles are useful for near vision, distance vision, protection and fashion. About 13 % of the participants reported that spectacles are useful only for protection. Less than 3 % of the participants reported that spectacles are useful for only near vision. This result is lower than study done in India (90 %) [12]. This might be due to the sample population in the Indian study was old people (mean age 45 years) who were more likely to be presbyopic and likely to use spectacles for near vision.

In this study 715 (91.7 %) of the respondents had favorable attitude towards spectacles use which is higher than a study reported from Nigeria (61.6 %) [14]. This might be due to cultural difference (beliefs of spectacles are not useful is much higher in Nigeria 60.53 % than this study area (14.7 %), whereas others beliefs towards spectacles were comparable).

Seven hundred and five (90.4 %) respondents intend to use spectacles if prescribed for them (including those who have been using spectacles and claiming that they will use it again). This result is higher than that of a study conducted in Nigeria, 122 (61.6 %) [14]. It might be due to two reasons: cultural difference (beliefs- don’t like spectacles was higher in Nigeria) and other options availability that can replace spectacles such as contact lens in Nigeria, and the inclusion of already spectacles users in this study.

Seventy five (9.6 %) of the respondents were not willing to wear spectacles if prescribed for them. This result is lower than study done in Nigeria (38.38 %) [14]. This might be due to numbers of study participants in Nigeria who responded “do not like spectacles” 38 (38.4 %), is higher than in this study 11 (14.6 %). The others reasons mentioned for not using were; social unacceptability 13 (17.3 %) and worsen the problem 15 (20.0 %) in this study which is comparable with study result reported from Nigeria. Ignorance 9 (29.0 %) and intolerance 3 (9.7 %) were other reasons for not using spectacles which is similar with the study reported from Nigeria [15].

One hundred and ninety four (24.9 %) of the respondents were spectacles users during the study period which is similar with the finding in India [13]. But this result is lower than a study reported from Nigeria [14]. This could be attributable to a different in refractive errors prevalence and accessibility to spectacles.

Among 194 (24.9 %) of spectacle users, 100 (51.6 %) of them use spectacles for protection and fashion. One hundred and nine (56.2 %) of spectacles users did not examine their eyes for spectacles use. Ninety eight (50.5 %) of spectacles users got their spectacles on streets and shops. The reason why more than half of the spectacles users did not get their eye examined and bought spectacles from street and shop might be due to their beliefs that all spectacles are protective and don’t need eye examination. Sixty five (33.5 %) of spectacles users use for protection which is much higher than study result from Nigeria. This might be due to adequate knowledge about spectacles and dusty as well as sunny environment in this study area.

Overall, 129 (66.5 %) of the spectacles users had poor practice of spectacles use. This might be related to getting spectacles from shops and on streets without their eyes examined. This result is almost similar with the study reported from Nigeria [15].

Sex, marital status, educational status, occupations, knowledge and attitude of the study participants about spectacles have no statistical significant association with practice of spectacles use. However, age of the respondent showed significant statistical association with practice of spectacles use. Age category 35–44 years (AOR = 0.20, 95 % CI; 0.04–0.93), 55–64 years (AOR = 0.18, 95 % CI; 0.04–0.84), and 65–74years (AOR = 0.02, 95 % CI; 0.00–0.21) had significant association with spectacles use.

Participants with primary school level of education (AOR: 2.79, 95 % CI 1.20–6.50) were about three times more likely to have adequate knowledge about spectacles. This might be due to participants with primary level education feel that they know everything.

Housewife (AOR = 3.40, 95 % CI; 1.35–8.54), participants who were unable to read and write (AOR: 3.51, 95 % CI 14–10.72) and inadequate knowledge about spectacles (AOR: 8.25 95 % CI 4.33–15.73) were more likely to have favorable attitude towards spectacles use. In this study, most of the participants were housewives and were not enable to read and write. The probability of having favorable attitude towards spectacle use might be due adherence to advice given to them and/or believing that spectacles are prescribed as mechanism to correct their eye problem.

The probability of having favorable attitude in those who have inadequate knowledge about spectacles might be due to their beliefs that spectacles are like medicine and they only given to individual for particular reasons which enhance the vision or confidence of them. Another possible reason might be ignorance from those who have adequate knowledge about spectacles.

Age category 35–44 years (AOR = 0.20, 95 % CI; 0.04–0.93), the likelihood of good practice of spectacles among this age category was less. This might be due to three reasons. Firstly, this age category is age of pre and early presbyopia. Though, this is the age at which probability of using spectacles for near vision starts due to pre and early presbyopia, they might use spectacles for limited reasons like for very small prints than as per prescribed to use. Secondly, they might have used spectacles bought from shops and on streets without their eye examined which might be too much or too low for their vision demand. This may result in poor adaptation or infrequent use of the spectacles. Thirdly, using spectacles, especially for near vision, might be assumed the sign of aging which might result in infrequent use of spectacles.

The likelihood of good practice of spectacles use among participants with age category 55–64 years (AOR = 0.18, 95 % CI; 0.04–0.84) and 65–74 years (AOR = 0.02, 95 % CI; 0.00–0.21) becomes less. The odds of good practice in the age groups also showed that there is a decreasing likelihood of using spectacles as age increases. This could be explained: first, spectacles bought from streets and shops without eye examination may not fulfill visual demand. Second, even though this age group is the time of presbyopia when near vision spectacles demanded, it is also the time of retirement so that they infrequently uses spectacles near vision. If they do not regularly checkup their eyes and do not change their spectacles, current spectacles may not satisfy their near vision demand. Third, because of regular spectacles change is needed to achieve their good near vision; there might be an economic problem to change their spectacles, which might lead to poor practice of spectacles use. Fourth, as age increases spectacles might not correct their vision to what they expected this is probably due to increase in age related ocular problems, which reduces correction of vision with spectacles. Hence, the likelihood of good practice of spectacles use among these age groups was minimal.

## Conclusion

In general, finding from this study showed that adult population in Gondar town have adequate knowledge and favorable attitude towards spectacles use. However, the practice of spectacles use is poor and old age people have poor practice of spectacles use. People with primary education level were more likely knowledgeable about spectacles use. Housewives and people who can’t read and write had favorable attitude towards spectacles use.

## Appendix

### Sample size determination and sampling procedure

Sample size was determined by using the single population proportion formula:

$$ \mathrm{n}=\frac{{\mathrm{z}}^2\upalpha /2\mathrm{p}\left(1-\mathrm{p}\right)}{{\mathrm{w}}^2}=\frac{(1.96)^2(0.5)\left(1-0.5\right)}{(0.05)^2}=384 $$

Where:

- α (Level of significance) = 5 %
- Z = value of z statistic at 95 % confidence level = 1.96
- W = maximum allowable error = 5 %
- P (proportion people with adequate knowledge, favorable attitude and good practice towards spectacle use) = 50 %
- q = 1–p = 50 %
- n = sample size

Considering a design effect of 2 for multistage sampling and 10 % of non-response rate the final sample size was 845 adults.
